# Evaluating methodological quality of prognostic prediction models on patient-reported outcome measurements after total hip and total knee arthroplasty: a systematic review

**DOI:** 10.1302/2633-1462.71.BJO-2025-0014.R1

**Published:** 2026-01-23

**Authors:** Jia Ye Lin, Pragadesh Natarajan, Victor King Liu, Deanne E. Jenkin, Wei-Ju Chang, Justine Naylor, Sam Adie

**Affiliations:** 1 St. George and Sutherland Centre for Clinical Orthopaedic Research (SCORe), Kogarah, Australia; 2 School of Clinical Medicine, UNSW Medicine & Health, St. George & Sutherland Clinical Campuses, University of New South Wales, Sydney, Australia; 3 Centre for Pain IMPACT, Neuroscience Research Australia (NeuRA), Randwick, Australia; 4 School of Health Sciences, Faculty of Health and Medicine, University of New South Wales, UNSW Sydney, Australia; 5 School of Clinical Medicine, UNSW Medicine & Health, South Western Sydney Clinical School, University of New South Wales, Sydney, Australia; 6 Ingham Institute for Applied Medical Research, Liverpool, Australia

**Keywords:** Total hip arthroplasty, total knee arthroplasty, prediction model, systematic review, total hip and total knee arthroplasty, patient-reported outcome measures (PROMs), Prognosis, MEDLINE, CINAHL, univariate analyses, joint arthroplasty, cancer, Knee Society Score

## Abstract

**Aims:**

Predictive modelling studies are increasingly popular, but the reporting quality in developing and validating these models remains suboptimal. This review aimed to evaluate the methodological quality of predictive models for patient-reported outcomes following total hip arthroplasty (THA) and total knee arthroplasty (TKA), identifying gaps in reporting and biases.

**Methods:**

The review followed PRISMA guidelines, appraising studies that developed and/or validated multivariate predictive models. Methodological quality was assessed using the Prediction model Risk Of Bias ASsessment Tool (PROBAST) tool, and reporting quality was evaluated using Transparent Reporting of a multivariable prediction model for Individual Prognosis Or Diagnosis (TRIPOD) guidelines. An electronic search was conducted across MEDLINE, EMBASE, and CINAHL up to 29 May 2025 and several studies from expert recommendation. Studies involving adults (aged ≥ 18 years) undergoing elective primary or revision THA or TKA were included, while univariate analyses and literature reviews were excluded.

**Results:**

The search identified 6,194 results, with 3,793 unique articles. A total of 58 studies were screened, and 41 were included. TRIPOD compliance ranged from 58% to 68%. Overall, 98% of studies had a low risk of bias in participant selection, but 83% showed a high risk of bias in analysis. Applicability concerns were low in 93% of studies.

**Conclusion:**

The review reveals significant methodological limitations in predictive models for THA and TKA outcomes, especially in analysis. Improving adherence to reporting guidelines is essential for enhancing transparency and reliability, ultimately supporting better clinical decision-making and patient outcomes.

Cite this article: *Bone Jt Open* 2026;7(1):115–129.

## Introduction

Osteoarthritis (OA) affects 9.3% of the population and over 30% of those aged > 65 years in Australia.^1^ Total hip arthroplasty (THA) and total knee arthroplasty (TKA) surgeries are effective for treating end-stage hip and knee OA. However, despite advances in implant technology, prosthesis materials, and surgical technique,^2^ a proportion of patients continue to report unsatisfactory outcomes, persistent pain, or poor function following THA (approximately 5% to 10%) and TKA (approximately 15% to 35%).^3-7^ Even after a technically well performed surgery, with normal examination and imaging, patients may not be relieved of symptoms of pain, stiffness, or disability.^8,9^ Unsatisfactory postoperative outcomes may reduce quality of life and predispose patients to revision surgery and higher healthcare costs.^10,11^ Therefore, identifying individuals who may not respond to THA or TKA can help orthopaedic surgeons to offer surgery to those most likely to benefit.

The concept of predictive modelling to support medical decision-making in individual patients is well established with commonly used risk-stratification tools such as CHA_2_DS_2_VASc, Well’s, and Model for End-Stage Liver Disease (MELD) scores.^12-14^ Good-quality predictive models are essential tools to identify patients who are at risk of developing certain conditions (diagnostic models), and the outcomes of treatment interventions (prognostic models). Indeed, the popularity of predictive modelling studies is increasing with improving AI modelling capabilities, however the reporting quality of research in developing and validating prediction models remains suboptimal.^15^ Substantial efforts have been made to improve methodological rigour and research transparency in this field,^13^ and guidelines such as the Transparent Reporting of a multivariable prediction model for Individual Prognosis Or Diagnosis (TRIPOD) statement and Checklist for critical Appraisal and data extraction for systematic Reviews of prediction Modelling Studies (CHARMS) checklist have been developed as a framework for reporting predictive modelling studies.^16^^17,18^ Systematic reviews on the methodological quality of prognostic models have been performed in common conditions such as cardiovascular disease,^19^ COVID-19,^20^ and cancer,^21^ but have not been evaluated in joint arthroplasty, despite a considerable body of literature directed at patient selection and optimization in joint arthroplasty and risk factors for poor postoperative outcomes.^22^ Little is known about the methodological quality, validity, and thus applicability of studies of prediction models for patient-reported outcomes after THA and TKA.

Published prognostic models in joint arthroplasty predict a heterogeneous range of outcomes, from perioperative complications and discharge destination,^23-26^ to long-term postoperative outcomes including pain,^27,28^ infection,^29-32^ readmission,^33^ revision,^34^ patient-reported function,^35,36^ range of motion,^37^ and satisfaction.^38^ However, shortcomings in study design, analysis, and reporting may increase the risk of bias, which ultimately affects the models’ predictive performance and applicability in clinical practice.^39,40^ A lack of methodological and analytic rigour may deter the integration of these studies into clinical guidelines and decision-making. Thus, a critical evaluation of the methodological quality of prediction modelling studies in THA and TKA is needed to ensure their clinical usefulness.

This systematic review aimed to: evaluate and report the quality of risk stratification and prediction modelling studies that predict patient-reported outcomes after THA and TKA; identify areas of methodological deficit and provide recommendations for future research; and synthesize the evidence on prediction models associated with postoperative patient-reported outcomes after THA and TKA surgery.

## Methods

This systematic review was prepared according to the PRISMA guidelines.^41,42^ The PRISMA checklist is provided in the Supplementary Material. The protocol of this systematic review was prospectively registered with the International Prospective Register of Systematic Reviews (PROSPERO, registration number CRD42021271828) and has been published.^43^

### Eligibility criteria

The PICOTS (Population, Intervention, Comparison, Outcome, Timing, Setting) approach was used to develop the eligibility criteria that was used to select relevant studies.^17,44^

Studies including adults aged 18 years or older undergoing elective THA or TKA were included. The surgeries were primary or revision THA/TKA, and either unilateral or bilateral joint arthroplasty. No restriction was placed on sex or race. Studies including participants receiving partial/hemi-replacements, or THA/TKA indicated for acute fracture, were excluded.

We evaluated prospective studies that used multivariate predictive statistical models to assess preoperative risk factors for predicting patient-reported outcome after THA or TKA. We included the following studies: 1) prediction model development studies without external validation of independent data; 2) prediction model development studies with external validation of independent data; 3) external model validation studies; and 4) studies updating a previously developed prediction model.

Eligible studies presented at least one formal prediction model or regression equation in such a way that it allowed calculation of the risk of poor postoperative outcome.

Included studies employed patient-reported outcome measures (PROMs) as the primary prediction outcome. As there is no single validated, reliable, and responsive PROM specifically for TKA or THA, we included prediction models using instruments to measure minimal clinically important difference in any patient-reported outcomes.^45^ These instruments include generic (quality of life) questionnaires such as the 36 or 12-Item Short-Form Health Surveys (SF-36 or SF-12)^46,47^ and the EuroQol five-dimension questionnaire (EQ-5D),^48^ or joint-specific questionnaires such as the Knee Society Score,^49^ the Western Ontario and McMaster Universities Arthritis Index,^50^ Oxford Knee Score,^51,52^ Oxford Hip Score,^52,53^ or Hip disability and Osteoarthritis Outcome Score.^54^ Studies reported a final prediction model(s) that only includes preoperative predictor variables.

The following types of study were excluded: 1) univariate prediction studies reporting bivariate associations between specific baseline clinical risk factors and postoperative PROMs, without multivariate adjustment for other sociodemographic or clinical parameters; 2) studies only identifying predictors associated with a PROM without an attempt to develop a prediction model; 3) studies that only predict non-PROM postoperative outcomes such as adverse events, complication rates, revision, falls, or clinician-assessed/reported outcomes; and 4) literature reviews and grey literature such as reports, conference abstracts, opinions, editorials, commentaries, and letters.

### Search strategy

To identify relevant studies, an electronic literature search of MEDLINE, EMBASE, and CINAHL was carried out on 29 May 2025. In addition to the predefined search strategy, we reviewed several studies from expert recommendation. Available published search filters were adapted and combined with medical subject headings (MeSH) and related free-text words for a sensitive yet specific search strategy. A combination of different keywords for THA or TKA and prediction model were used to identify relevant literature. The search strategies were tailored to each database. No restriction was placed on the publication period up until search date of 29 May 2025. Only articles in the English language were included. If non-English studies had an English abstract, they were included in the title and abstract screening, but excluded from full-text screening. The reference lists of included studies and existing relevant reviews was also screened for potentially relevant studies. References were also searched for the original prediction model development study in cases of external model updating and recalibration.

### Study selection

The complete references of the studies retrieved from the above search strategy were exported into Endnote X9 and duplicates removed. Three reviewers (PN, VKL, JYL) independently assessed the title and abstract of all studies identified through the search against the eligibility criteria. The full text of all eligible studies was retrieved. Disagreements on study eligibility were resolved by consensus and a third reviewer (WJC) consulted for arbitration as needed. Search results and reasons for excluded articles at each stage of study selection was documented and reported in a PRISMA flowchart.^55^

### Data extraction

Three reviewers independently (PN, VKL, JYL) conducted the data extraction from the final list of eligible studies. Any disagreements in the extracted data were resolved through discussion with an additional reviewer (WJC). A piloting phase was introduced before the formal data extraction, where two randomly chosen articles from the eligible articles were used to test a piloted data extraction spreadsheet and the definitions of the items to be collected. Disagreements were discussed to achieve consensus and modifications to the piloted spreadsheet were made. This customized data extraction spreadsheet was then reviewed and agreed by all the reviewers before its use in the formal data extraction.

We collected information in the domains related to prediction modelling adapted from the CHARMS and TRIPOD statements.^17,18,56^ The following information was extracted from the eligible studies: study characteristics, participants, outcome measures, predictor variables, model characteristics (including sample size), missing data, model development, model performance, model evaluation, multivariable model result, and/or alternative final prediction models.^57^

### Study quality and risk of bias

The methodological quality of the included studies was assessed by two reviewers independently with disagreements resolved by consensus. The risk of bias and applicability concerns was assessed using the PROBAST (Prediction model Risk of Bias Assessment Tool) in four domains of participants, predictors, outcome, and analysis (a total of 20 signalling questions) for the development and validation of prediction models.^58^ Signalling questions were rated (yes, probably yes, probably no, no, or no information) to help make judgement for risk of bias as ‘high’, ‘low’, or ‘unclear’ for each domain. Applicability concerns of three domains of participants, predictors, and outcome were rated (high/low/unclear). Overall risk of bias for each prediction model was assessed across all four domains based on the following criteria: low, high, and unclear. Low: when all domains rated as low risk of bias; a prediction development model without external validation based on a very large dataset and included internal validation. High: when at least one domain rated as high risk of bias; a prediction development model without internal or external validation rated as low risk of bias. Unclear: when at least one domain rated as unclear risk of bias and the rest of the other domains as low risk of bias. Similarly, overall applicability concerns for each model was also assessed across three domains: low, high, or unclear.

### Data synthesis

A narrative review was conducted to synthesize the evidence for the risk of bias and applicability concerns of the prediction modelling studies. Data of the selected studies were tabulated or categorized in the following domains: study characteristics, outcomes, predictors, methodological findings, model performance, methodological quality.

All domains related to methodological quality were assessed. Specifically, the usefulness and overall applicability of the prediction models was described. The risk of bias and applicability concerns were reported as counts and percentages to underline the most critically affected domains of bias and applicability.

A meta-analysis was initially planned; however, no two included studies assessed the predictive performance (discrimination and calibration) of the models on the same PROM with sufficient available data.

## Results

### Study characteristics

**Fig. 1:**
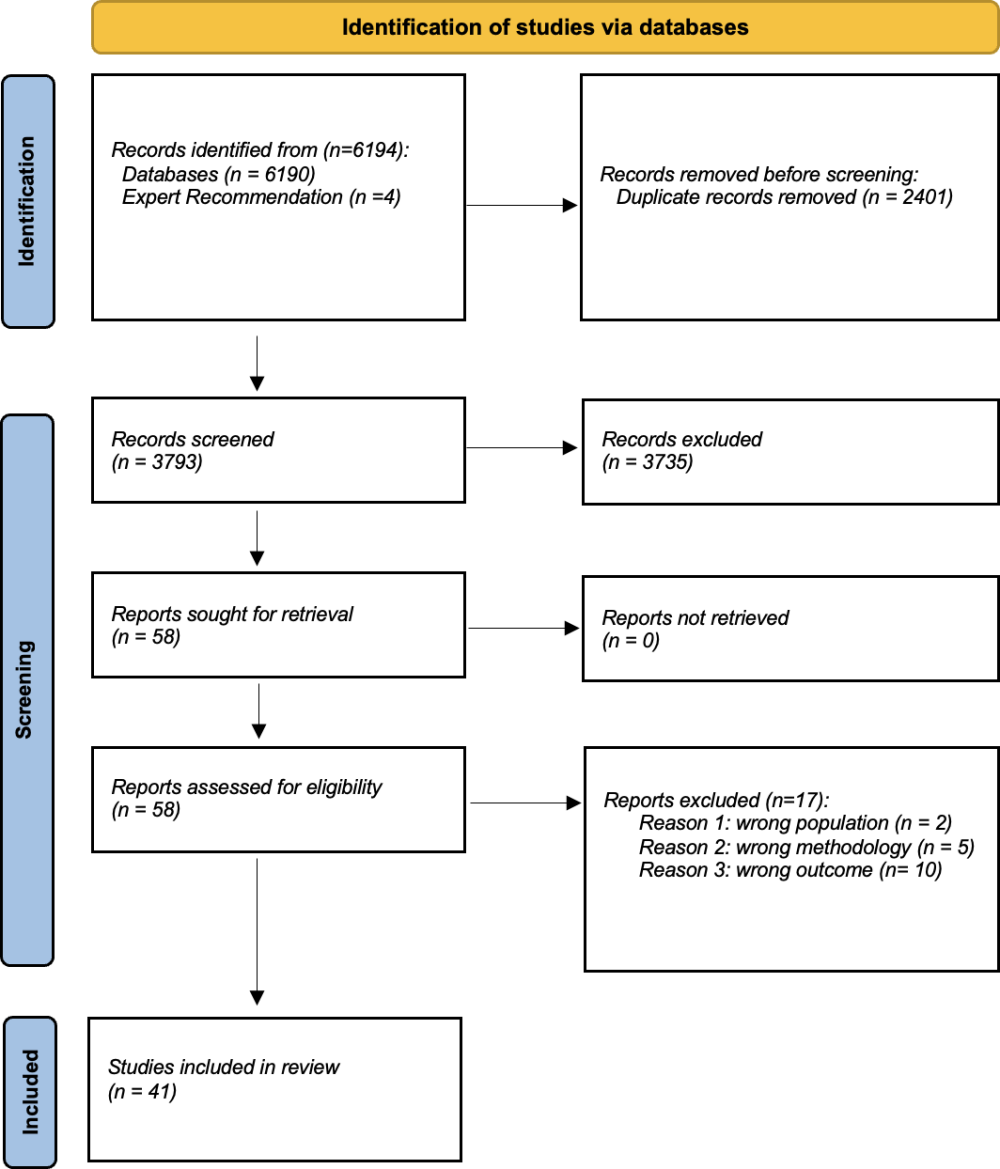
PRISMA 2020 flow diagram for new systematic reviews which included searches of databases and registers only. Reason 1: Is the population adults aged ≥ 18 years and received selective total hip arthroplasty or total knee arthroplasty (either primary or revision; unilateral or bilateral)? Excluded if: 1) partial or hemireplacement or 2) due to acute fracture. Reason 2: Did the study use multivariate predictive modelling approach examining preoperative predictor variables? Studies must present a prediction model or a regression equation that allows for risk calculation. Reason 3: Did the study use patient-reported outcome measures (PROMs) as the primary prediction outcome? Study must use PROMs as the primary prediction outcome.

The predefined search identified 6,194 results with 3,793 unique articles, including four articles identified through expert recommendation. Overall, 58 studies were eligible for full-text screening and 41 studies were included in the final analysis, as shown in the PRISMA diagram (Figure 1). The articles were classified according to the aims of the research: 30 (73%) as model development only,^4,27,35,38,59-84^ eight (20%) as model development as well as validation of the same model,^28,85-91^ and three (7%) as external validation only.^92-94^ There are 13 studies that investigated unilateral THA, 26 unilateral THA, one study investigated primary unilateral TKA and THA, and one study investigated both unilateral and bilateral TKA and THA. A total of 12 studies were prospective cohort studies and 29 were retrospective cohorts. The mean patient age across all studies ranged from 52 to 74 years, and the proportion of females ranged from 7% to 80%. Further characteristics for the included studies are given in Table I.

**Table I.** Characteristics for the included studies.

Study	Data source	Type of surgery	Type of study	Number of patients	Mean age, yrs	Female, %	PROM	Timing of outcome measurement
Berliner et al, 2016^59^	Single institution registry data	Primary unilateral THA	Development	537	62	60	SF-12v2, HOOS	Preop, 1 yr postop
Berliner et al, 2017^60^	Single institution registry data	Primary unilateral TKA	Development	562	67	60	SF-12v2, KOOS	Preop, 1 yr postop
Clement et al, 2021^61^	Single institution registry data	Primary unilateral TKA	Development	3,324	69.1	54	WOMAC	Preop, 1 yr postop
Dowsey et al, 2016^4^	Single institution registry data	Primary unilateral TKA	Development	615	68.9	64	SF12	Preop, 3 mths, 1 yr postop
Farooq et al, 2020^62^	Single institution registry data	Primary unilateral TKA	Development	897	66.1	73	Likert Scale	Preop, 1 yr postop
Harris et al, 2021^63^	Cohort study	Primary unilateral TKA	Development	587	66.1	7	KOOS	Preop, 1 yr postop
Judge et al, 2012^64^	Health district registry data	Primary unilateral THA	Development	249	67.2	64	SF-36	Preop, 8.5 yrs postop
Katakam et al, 2022^65^	Multiple institutions registry data	Primary unilateral TKA	Development	744	68	61	KOOS	Preop, 1 yr postop
Kunze et al, 2020^66^	Multiple institutions registry data	Primary unilateral TKA	Development	430	66.2	68	KSS	Preop, 2 yr postop
Lungu et al, 2014^35^	Multiple institutions registry data	Primary unilateral TKA	Development	141	66	66	WOMAC	Preop, 6 mths postop
Lungu et al, 2015^67^	Multiple institutions registry data	Primary unilateral THA	Development	265	52	34	WOMAC	Preop, 1 to 2 yrs postop
Nemes et al, 2018^68^	National registry data	Primary unilateral THA	Development	6,460	69.2	58	EQ-5D	Preop, 1 yr postop
Pronk et al, 2021^69^	Single institution registry data	Primary unilateral TKA	Development	1,196	65.3	53	OKS, KOOS, EQ-5D	Preop, 1 yr postop
Pua et al, 2016^70^	Cohort study	Primary unilateral TKA	Development	1,096	67	75	OHS	Preop, 6 mths postop
Pua et al, 2019^71^	Cohort study	Primary unilateral TKA	Development	4,206	68	71	OKS	Preop, 6 mths postop
Rogers et al, 2015^72^	Multiple institutions registry data	Primary unilateral THA	Development	446	63	51	WOMAC	Preop, 1 yr postop
Shim et al, 2018^27^	Cohort study	Primary unilateral TKA	Development	721	68.6	53	OKS	Preop, 6 mths postop
Sniderman et al, 2021^73^	Cohort study	Primary unilateral THA	Development	160	66.7	44	HOOS	Preop, 3 mths postop
Tanaka et al, 2021^74^	Multiple institutions registry data	Primary unilateral TKA	Development	116	73.9	80	JKOM	Preop, 3 mths postop
Van Onsem et al, 2016^38^	Single institution registry data	Primary unilateral TKA	Development	113	65.2	56	KOOS, OKS, EQ-5D	Preop, 3 mths postop
Vissers et al, 2020^75^	Single institution registry data	Primary unilateral TKA	Development	569	68.9	58	OKS, KOOS	Preop, 6 mths postop
Wang et al, 2010^76^	Single institution registry data	Primary unilateral THA	Development	97	61.65	65	WOMAC	Preop, 1 yr postop
Weber et al, 2019^77^	Cohort study	Primary unilateral THA	Development	126	61.6	41	SF-36, HOOS, EQ-5D	Preop, 1 yr postop
Aggarwal et al, 2022^78^	Single, registry data	Primary unilateral THA	Development	1,412	67	53.3	HOOS	Preop, 6 mths postop
Zhou et al, 2023^79^	Single, registry data	Primary unilateral TKA	Development	3,755	69.7	64.8	HRQoL	Preop, 12 mths postop
Verullens et al, 2025^80^	Single, cohort study	Primary unilateral TKA	Development	223	65.52	49.8	KOOS	Preop, 12 mths postop
Zhang et al, 2021^81^	Single, cohort study	Primary unilateral THA	Development	1,508	62.9	69.8	SF-36, WOMAC, OHS	Preop, 2 yrs postop
Strahl et al, 2025^82^	Single, cohort study	Primary unilateral TKA	Development	236	68.8	59.7	KSS, KOOS, VAS, PHQ-4	Preop, 1 mth and 1 yr postop
Dowsey et al, 2022^83^	Single, registry data	Primary unilateral THA	Development	2,177	66.5	56	WOMAC	Preop, 1 yr postop
Langenberger et al, 2023^84^	Single, registry data	Primary unilateral THA and TKA	Development	3,389	66	N/A	EQ-5D-5L, EQ-VAS, HOOS	Preop, 1 yr postop
Bloomfield et al, 2021^85^	Cohort study	Primary unilateral TKA	Both	68	67.5	50	KSS, UCLA activity score	Preop, 3, 6, 12 mths postop
Fontana et al, 2019^86^	Single institution registry data	Primary unilateral/bilateral TKA and THA	Both	13,719	64.8	56	SF-36, HOOS, KOOS	Preop, 2 yrs postop
Garriga et al, 2018^87^	Cohort study	Primary unilateral TKA	Both	450	70	58	WOMAC	Preop, 1 yr postop
Liu et al, 2021^88^	Single institution registry data	Primary unilateral TKA	Both	545	72.2	75	KSS, WOMAC, SF-12	Preop, 1 yr postop
Sanchez-Santos et al, 2018^28^	Multiple institutions registry data	Primary unilateral TKA	Both	1,649	N/A	56	OKS, EQ-5D	Preop, 1 yr postop
Stockli et al, 2014^89^	Cohort study	Primary unilateral THA	Both	375	70	50	WOMAC	Preop, 3 mths postop
Tolk et al, 2020^90^	National registry data	Primary unilateral TKA	Both	7,071	68.4	63	OKS, KOOS, EQ-5D	Preop, 1 yr postop
Twiggs et al, 2019^91^	Multiple institutions registry data	Primary unilateral TKA	Both	150	65.7	53	KOOS	Preop, 1 yr postop
Calkins et al, 2019^92^	Cohort study	Primary unilateral TKA	Validation	145	64.9	59	SF-12, KOOS Jr	Preop, 3 mths postop
Pulik et al 2020^93^	Single institution registry data	Primary unilateral THA	Validation	365	65.11	57	WOMAC, HKASS	Preop, 3 yrs postop
Riddle et al, 2017^94^	Multiple institutions registry data	Primary unilateral TKA	Validation	427	68.24	60	WOMAC	Preop, 536 days postop

ED-5D, EuroQol five-dimension questionnaire; HKASS, Hip and Knee Arthroplasty Satisfaction Scale; HOOS, Hip disability and Osteoarthritis Outcome Score; JKOM, Japanese Knee Osteoarthritis Measure; KOOS, Knee disability and Osteoarthritis Outcome Score; KSS, Knee Society Score; OHS, Oxford Hip Score; OKS, Oxford Knee Score; postop, Post-operation; Preop, Pre-operation; PROM, patient-reported outcome measure; SF-36, 36-Item Short-Form Health Survey; SF-12v2, 12-Item Short-Form (version 2) Health Survey; THA, total hip arthroplasty; TKA, total knee arthroplasty; UCLA, University of California, Los Angeles; VAS, visual analogue scale; WOMAC, the Western Ontario and McMaster Universities Arthritis Index.

### Reporting quality: compliance with the TRIPOD guideline

**Fig. 2:**
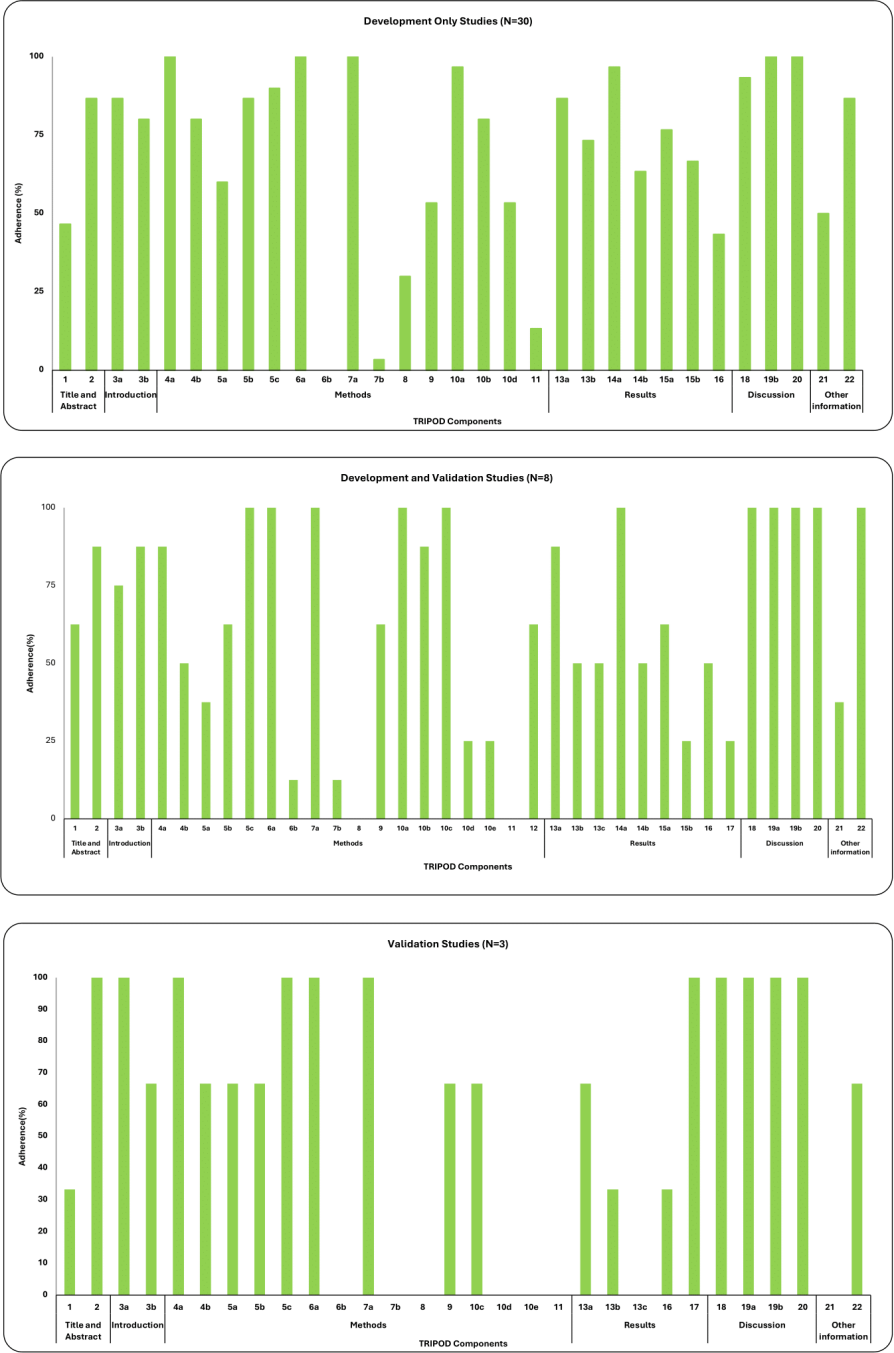
a) Reporting of Transparent Reporting of a multivariable prediction model for Individual Prognosis Or Diagnosis (TRIPOD) items in development-only studies (n = 30). Adherence is defined as the proportion of studies that fulfilled each TRIPOD checklist item. Bars represent the percentage of studies reporting each item in accordance with TRIPOD guidance. Only applicable TRIPOD items were included. b) Reporting of TRIPOD items in development and validation studies (n = 8). Adherence is defined as the proportion of studies that fulfilled each TRIPOD checklist item. Bars represent the percentage of studies reporting each item in accordance with TRIPOD guidance. Only applicable TRIPOD items were included. c) Reporting of TRIPOD items in validation only studies (n = 3). Adherence is defined as the proportion of studies that fulfilled each TRIPOD checklist item. Bars represent the percentage of studies reporting each item in accordance with TRIPOD guidance. Only applicable TRIPOD items were included.

Fulfilment of TRIPOD guideline components ranged from in 64% in studies of both model development and validation (n = 8), 70% in studies of development only (n = 30), and 58% in studies of validation only (n = 3). Poorly reported items (< 25% of included studies) were items 6b, 7b, and 11 in studies of development only (Figure 2a), items 6b, 7b, 8, and 11 in studies of both development and validation (Figure 2b), and items 6b, 7b, 8, 10d, 10e, 11, 13c, and 21 in studies of external validation only (Figure 2c). These items corresponded to sections on reporting outcomes (in particular, blinded assessment), statistical analysis, and use of risk groups for stratifying predictions. Notably, validation-only studies reported poorly on model performance, model updating, comparing validation data with development data, and providing appropriate supplementary resources (such as datasets). However, this was a limited sample size (n = 3). Other moderately (25% to 75% of included studies) and well reported items (> 75% of included studies) are further detailed in Table II.

**Table II.** Reporting quality of included studies by Transparent Reporting of a multivariable prediction model for Individual Prognosis Or Diagnosis guidelines.

Section	Item	Description	Development and validation (n = 8)	Development only (n = 30)	Validation (n = 3)
Title	1	Identify the study as developing and/or validating a multivariable prediction model, the target population, and the outcome to be predicted	Fair	Fair	Fair
Abstract	2	Provide a summary of objectives, study design, setting, participants, sample size, predictors, outcome, statistical analysis, results, and conclusions	Good	Good	Good
Background and Objectives	3a	Explain the medical context (including whether diagnostic or prognostic) and rationale for developing or validating the multivariable prediction model, including references to existing models	Fair	Good	Good
	3b	Specify the objectives, including whether the study describes the development or validation of the model, or both	Good	Good	Fair
Source of data	4a	Describe the study design or source of data (for example, randomized trial, cohort, or registry data), separately for the development and validation datasets, if applicable	Good	Good	Good
	4b	Specify the key study dates, including start of accrual; end of accrual; and, if applicable, end of follow-up	Fair	Good	Fair
Participants	5a	Specify key elements of the study setting (for example, primary care, secondary care, general population) including number and location of centres	Fair	Fair	Fair
	5b	Describe eligibility criteria for participants	Fair	Good	Fair
	5c	Give details of treatments received, if relevant	Good	Good	Good
Outcome	6a	Clearly define the outcome that is predicted by the prediction model, including how and when assessed	Good	Good	Good
	6b	Report any actions to blind assessment of the outcome to be predicted	Poor	Poor	Poor
Predictors	7a	Clearly define all predictors used in developing the multivariable prediction model, including how and when they were measured	Good	Good	Good
	7b	Report any actions to blind assessment of predictors for the outcome and other predictors	Poor	Poor	Poor
Sample size	8	Explain how the study size was arrived at	Poor	Fair	Poor
Missing data	9	Describe how missing data were handled (for example, complete-case analysis, single imputation, multiple imputation) with details of any imputation method	Fair	Fair	Fair
Statistical analysis	10 a	Describe how predictors were handled in the analyses	Good	Good	N/A
	10b	Specify type of model, all model-building procedures (including any predictor selection), and method for internal validation	Good	Good	N/A
	10 c	For validation, describe how the predictions were calculated	Good	N/A	Fair
	10d	Specify all measures used to assess model performance and, if relevant, to compare multiple models	Fair	Fair	Poor
	10e	Describe any model updating (for example, recalibration) arising from the validation, if done	Fair	N/A	Poor
Risk groups	11	Provide details on how risk groups were created, if done	Poor	Poor	Poor
Development vs Validation (dataset)	12	For validation, identify any differences from the development data in setting, eligibility criteria, outcome, and predictors	Fair	N/A	N/A
Participants	13 a	Describe the flow of participants through the study, including the number of participants with and without the outcome and, if applicable, a summary of the follow-up time. A diagram may be helpful	Good	Good	Fair
	13b	Describe the characteristics of the participants (basic demographic details, clinical features, available predictors), including the number of participants with missing data for predictors and outcome	Fair	Fair	Fair
	13c	For validation, show a comparison with the development data of the distribution of important variables (demographic details, predictors, and outcome).	Fair	N/A	Poor
Model development	14a	Specify the number of participants and outcome events in each analysis	Good	Good	N/A
	14b	If done, report the unadjusted association between each candidate predictor and outcome	Fair	Fair	N/A
Model specification	15a	Present the full prediction model to allow predictions for individuals (that is, all regression coefficients, and model intercept or baseline survival at a given timepoint)	Fair	Good	N/A
	15b	Explain how to use the prediction model	Fair	Fair	N/A
Model performance	16	Report performance measures (with CIs) for the prediction model	Fair	Fair	Fair
Model updating	17	If done, report the results from any model updating (that is, model specification, model performance)	Fair	N/A	Good
Limitations	18	Discuss any limitations of the study (such as nonrepresentative sample, few events per predictor, missing data)	Good	Good	Good
Interpretation	19 a	For validation, discuss the results with reference to performance in the development data, and any other validation data	Good	N/A	Good
	19b	Give an overall interpretation of the results, considering objectives, limitations, results from similar studies, and other relevant evidence	Good	Good	Good
Implications	20	Discuss the potential clinical use of the model and implications for future research	Good	Good	Good
Other – Supplementary Information	21	Provide information about the availability of supplementary resources, such as study protocol, Web calculator, and datasets	Fair	Fair	Poor
Other – Funding	22	Give the source of funding and the role of the funders for the present study	Good	Good	Fair

Good (> 75%), Fair (75% to 25%), Poor (< 25%), N/A (development or validation only items).

N/A, not applicable.

### Methodological quality: compliance with the PROBAST tool

**Fig. 3:**
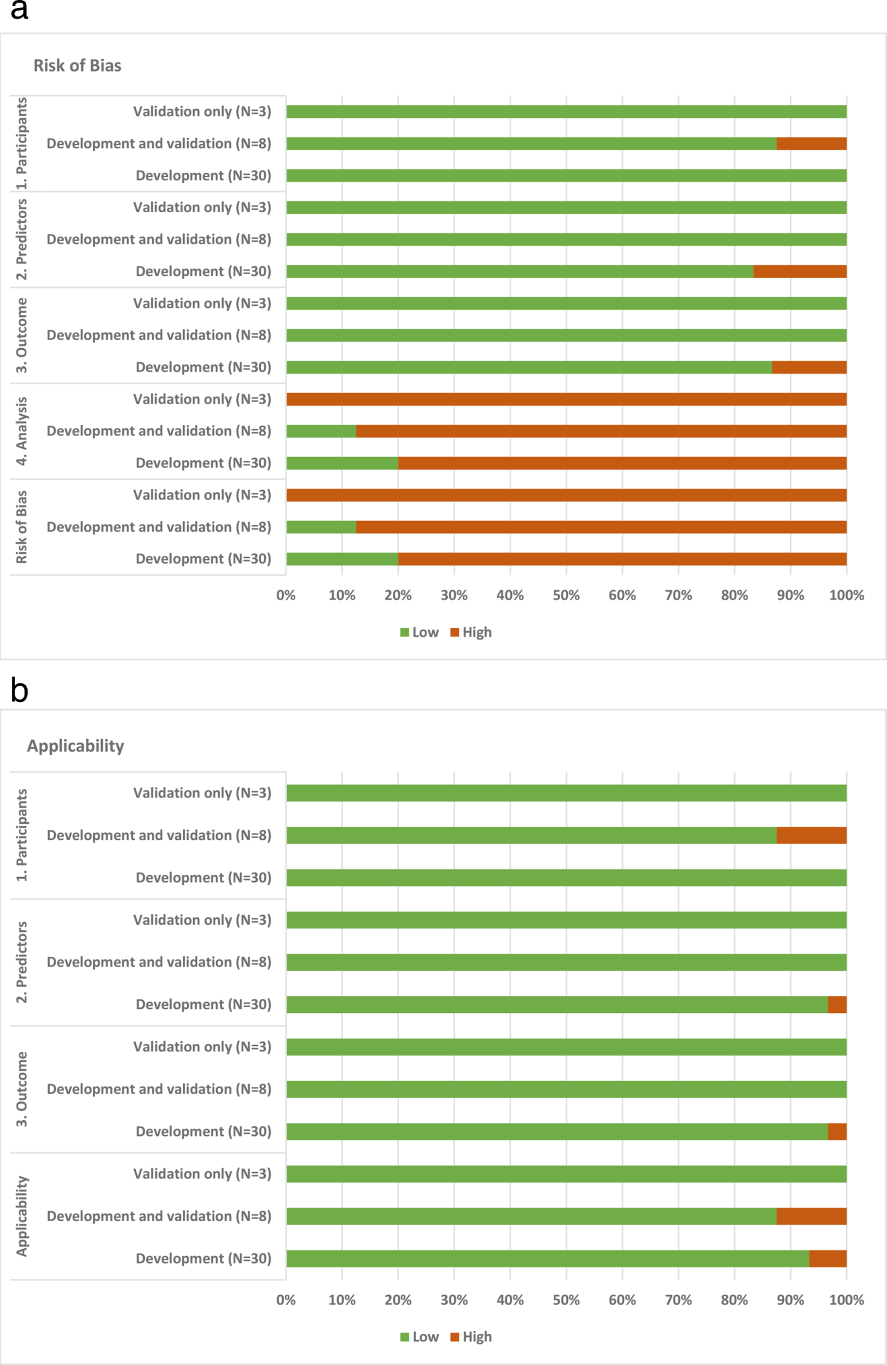
a) Prediction model risk of bias assessment tool assessment of included models for risk of bias (n = 41 for all included studies, n = 30 for development studies, n = 3 for validation studies, n = 8 for development and validation studies). b) Prediction model risk of bias assessment tool assessment of included models for concern for applicability (n = 41 for all included studies, n = 30 for development studies, n = 3 for validation studies, n = 8 for development and validation studies).

Risk of bias was generally low across included studies in the domains for participants (40/41, 98%), predictors (36/41, 88%), and outcomes (37/41, 90%) (Figure 3a). However, risk of bias was high in the analysis domain (35/41, 85%) and therefore overall there was a high risk of bias (36/41, 88%). Concern for applicability was generally low (38/41, 93%) across all included studies (Figure 3b).

## Discussion

This systematic review evaluated the methodological quality of predictive models for patient-reported outcomes after THA and TKA. We found that on average, only two-thirds of TRIPOD items were reported, indicating deficits in reporting quality. In terms of methodological quality, we also found that there were considerable limitations in the analysis section of predictive modelling studies when assessed with PROBAST criteria. Despite the increasing popularity of predictive modelling studies in the literature,^95^ there remains a deficit in reporting and methodological quality.^96^

To the best of our knowledge, this is the first assessment of methodological and reporting quality of studies on prediction models after TKA and THA. However, our findings align with broader trends observed in other areas of the medical field, where predictive models often face substantial challenges related to study design and statistical analysis. For instance, a systematic review by Wynants et al^20^ on predictive models for COVID-19 reported high risk of bias in newly developed and validation models with the analysis domain being the most problematic. Similarly, another by Gu et al^97^ focusing on gastric cancer prediction models highlighted that the high risk of bias in the analysis domain was due to poor methods being employed.^97^ This high risk of bias across the reviews, including ours, can largely be attributed to small sample sizes, which can heighten the risk of overfitting and optimism, as well as the use of inadequate statistical techniques and incomplete or improper validation techniques.^98^ Similar to our findings, incomplete data were common in other studies with almost 50% of studies having some form of missing data, which may create statistical bias.^20,97^ Excluding outliers in complete-case analysis can typically decrease the discriminatory power of multivariable models, while replacing them with imputation methods can also bias effect estimates, making the sample less representative.^99^

Systematic reviews assessing studies of development or validation of prediction models in other patient populations have reported similar findings to the present study. Mallett et al^100^ examined 47 published reports of new prognostic models for patient outcomes in cancer. Reporting was found to be poor, with insufficient information in all aspects of model development, from descriptions of patient data to statistical modelling methods. Bouwmeester et al^101^ evaluated 71 reports of all prediction studies published in 2008 in six high-impact general medical journals and likewise resulted in an overwhelmingly poor level of reporting. These findings suggest that across various patient populations, including in our study, there is generally a poor level of methodological and reporting quality of prediction model studies. There is a need for increased adoption of reporting quality tools such as TRIPOD and PROBAST, to standardize the development of predictive models, to enable valid comparisons to be made across datasets and generalizability to clinical practice.

This review follows a systematic approach and was preregistered, which improves its transparency, reduces the risk of bias, and ensures that the methods were defined a priori. This strengthens the credibility and reproducibility of our findings. The use of PROBAST and TRIPOD is widespread across numerous reviews, contributing to their validation as reporting and methodological guidelines. The TRIPOD is also recommended by the Equator Network; they have become ubiquitous and widely accepted in major journals.^102^ There is strong evidence that the reporting of published research has improved since the adoption of the EQUATOR Network guidelines, which supports the likelihood that TRIPOD will also be successful.^103^ This systematic review included prospective and retrospective cohort studies, with the majority being retrospective. The inclusion of both study types improves generalizability to the large proportion of retrospective registry-driven predictive models in joint arthroplasty.

In addition, the review’s strength lies in its systematic literature search, stringent selection strategy, and detailed extraction of key data on predictors, outcomes, and study populations adhering to PRISMA recommendations. However, one of the limitations of our study is the restriction of our search to English-language articles. Additionally, the heterogeneity of the included studies can be problematic as there can be variability in study designs, populations, and outcome measures, which can complicate data synthesis and interpretation of results.^104^ At the time of writing, updated guidance for methodological and reporting quality of predictive modelling studies is also being developed, incorporating AI model-specific domains (TRIPOD + AI and PROBAST + AI).^105,106^ Given that our review is an assessment of the overall reporting and methodological quality of studies in literature, we do not anticipate these trends across literature to change significantly with the addition of a few recent studies.

To improve the reporting and conduct of future predictive modelling studies, we suggest that the PROBAST tool is used at an early stage during the inception and design of these studies (with appropriate statistician input as required), and the TRIPOD reporting guidelines are rigorously adhered to – using a similar approach to how CONSORT and other reporting guidelines have been widely adopted.^19,103^ Enhancing the reliability of prediction models ultimately improves patient care in joint arthroplasty. Furthermore, journals should implement these guidelines and enforce compliance by authors before publication, ensuring higher standards of methodological rigour. This approach can help prevent the dissemination of poorly reported research, which may lead clinicians to make decisions based on inaccurate or incomplete information—potentially compromising patient outcomes if clinical management is not grounded in robust scientific evidence.^107^

In conclusion, our findings highlight significant methodological limitations in existing predictive models for THA and TKA outcomes. This emphasizes the need to improve methodological rigour in predictive modelling studies related to THA and TKA. Compliance with established reporting guidelines such as TRIPOD and PROBAST is essential to improve reporting and methodological rigour as well as research transparency in diagnostic and prognostic predictive modelling, to ensure their applicability to clinical practice.

**Take home message**

- Poor methodological and reporting quality in predictive models for patient-reported outcomes after total hip arthroplasty and total knee arthroplasty can lead to inaccurate predictions, limiting their utility in guiding shared decision-making and personalized care.

- Improving adherence to TRIPOD and PROBAST standards is important to ensure reliable models that support evidence-based clinical practice and optimize patient outcomes.

## Data Availability

The datasets used and/or analyzed during the current study are available from the corresponding author on reasonable request.

## References

[1] Australian Institute of Health and Welfare Chronic musculoskeletal conditions: Osteoarthritis AIHW 2020 https://www.aihw.gov.au/reports/chronic-musculoskeletal-conditions/osteoarthritis date last accessed 23 December 2025

[2] Batailler C Swan J Sappey Marinier E Servien E Lustig S New technologies in knee arthroplasty: current concepts J Clin Med 2020 10 1 47 10.3390/jcm10010047 33375702 PMC7795103

[3] Gandhi R Davey JR Mahomed NN Predicting patient dissatisfaction following joint replacement surgery J Rheumatol 2008 35 12 2415 2418 10.3899/jrheum.080295 19004032

[4] Dowsey MM Spelman T Choong PFM Development of a prognostic nomogram for predicting the probability of nonresponse to total knee arthroplasty 1 year after surgery J Arthroplasty 2016 31 8 1654 1660 10.1016/j.arth.2016.02.003 26935945

[5] Singh JA Lewallen DG Predictors of activity limitation and dependence on walking aids after primary total hip arthroplasty J Am Geriatr Soc 2010 58 12 2387 2393 10.1111/j.1532-5415.2010.03182.x 21143444 PMC3850176

[6] Scott CEH Bugler KE Clement ND MacDonald D Howie CR Biant LC Patient expectations of arthroplasty of the hip and knee J Bone Joint Surg Br 2012 94-B 7 974 981 10.1302/0301-620X.94B7.28219 22733956

[7] Nilsdotter AK Toksvig-Larsen S Roos EM Knee arthroplasty: are patients’ expectations fulfilled? A prospective study of pain and function in 102 patients with 5-year follow-up Acta Orthop 2009 80 1 55 61 10.1080/17453670902805007 19234886 PMC2823230

[8] Wang X Ida M Uyama K Naito Y Kawaguchi M Persistent postoperative pain at 1 year after orthopedic surgery and its association with functional disability J Anesth 2023 37 2 248 253 10.1007/s00540-022-03156-2 36565365

[9] Haanstra TM van den Berg T Ostelo RW et al. Systematic review: do patient expectations influence treatment outcomes in total knee and total hip arthroplasty? Health Qual Life Outcomes 2012 10 152 10.1186/1477-7525-10-152 23245187 PMC3568025

[10] Weber M Renkawitz T Voellner F et al. Revision surgery in total joint replacement is cost-intensive Biomed Res Int 2018 2018 1 8 10.1155/2018/8987104 30356391 PMC6176320

[11] Maradit Kremers H Kremers WK Berry DJ Lewallen DG Patient-reported outcomes can be used to identify patients at risk for total knee arthroplasty revision and potentially individualize postsurgery follow-up J Arthroplasty 2017 32 11 3304 3307 10.1016/j.arth.2017.05.043 28648711

[12] Lip GYH Nieuwlaat R Pisters R Lane DA Crijns HJGM Refining clinical risk stratification for predicting stroke and thromboembolism in atrial fibrillation using a novel risk factor-based approach: the Euro Heart Survey on atrial fibrillation Chest 2010 137 2 263 272 10.1378/chest.09-1584 19762550

[13] Kamath PS Wiesner RH Malinchoc M et al. A model to predict survival in patients with end-stage liver disease Hepatology 2001 33 2 464 470 10.1053/jhep.2001.22172 11172350

[14] Wolf SJ McCubbin TR Feldhaus KM Faragher JP Adcock DM Prospective validation of Wells Criteria in the evaluation of patients with suspected pulmonary embolism Ann Emerg Med 2004 44 5 503 510 10.1016/j.annemergmed.2004.04.002 15520710

[15] Stahl D New horizons in prediction modelling using machine learning in older people’s healthcare research Age Ageing 2024 53 9 afae201 10.1093/ageing/afae201 39311424 PMC11417961

[16] Collins GS Reitsma JB Altman DG Moons KGM Transparent Reporting of a multivariable prediction model for Individual Prognosis or Diagnosis (TRIPOD): the TRIPOD statement Ann Intern Med 2015 162 1 55 63 10.7326/M14-0697 25560714

[17] Moons KGM Altman DG Reitsma JB et al. Transparent reporting of a multivariable prediction model for Individual Prognosis or Diagnosis (TRIPOD): explanation and elaboration Ann Intern Med 2015 162 1 W1 73 10.7326/M14-0698 25560730

[18] Moons KGM de Groot JAH Bouwmeester W et al. Critical appraisal and data extraction for systematic reviews of prediction modelling studies: the CHARMS checklist PLoS Med 2014 11 10 e1001744 10.1371/journal.pmed.1001744 25314315 PMC4196729

[19] Damen JAAG Hooft L Schuit E et al. Prediction models for cardiovascular disease risk in the general population: systematic review BMJ 2016 353 i2416 10.1136/bmj.i2416 27184143 PMC4868251

[20] Wynants L Van Calster B Collins GS et al. Prediction models for diagnosis and prognosis of covid-19: systematic review and critical appraisal BMJ 2020 369 m1328 10.1136/bmj.m1328 32265220 PMC7222643

[21] Deliu N Cottone F Collins GS Anota A Efficace F Evaluating methodological quality of Prognostic models Including Patient-reported HeAlth outcomes iN oncologY (EPIPHANY): a systematic review protocol BMJ Open 2018 8 10 e025054 10.1136/bmjopen-2018-025054 30361409 PMC6224737

[22] Adie S Harris I Chuan A Lewis P Naylor JM Selecting and optimising patients for total knee arthroplasty Med J Aust 2019 210 3 135 141 10.5694/mja2.12109 30656689

[23] Romine LB May RG Taylor HD Chimento GF Accuracy and clinical utility of a peri-operative risk calculator for total knee arthroplasty J Arthroplasty 2013 28 3 445 448 10.1016/j.arth.2012.08.014 23146586

[24] Wuerz TH Kent DM Malchau H Rubash HE A nomogram to predict major complications after hip and knee arthroplasty J Arthroplasty 2014 29 7 1457 1462 10.1016/j.arth.2013.09.007 24793891

[25] Harris AH Kuo AC Bowe T Gupta S Nordin D Giori NJ Prediction models for 30-day mortality and complications after total knee and hip arthroplasties for veteran health administration patients with osteoarthritis J Arthroplasty 2018 33 5 1539 1545 10.1016/j.arth.2017.12.003 29398261 PMC6508537

[26] Oldmeadow LB McBurney H Robertson VJ Predicting risk of extended inpatient rehabilitation after hip or knee arthroplasty J Arthroplasty 2003 18 6 775 779 10.1016/s0883-5403(03)00151-7 14513453

[27] Shim J Mclernon DJ Hamilton D Simpson HA Beasley M Macfarlane GJ Development of a clinical risk score for pain and function following total knee arthroplasty: results from the TRIO study Rheumatol Adv Pract 2018 2 2 rky021 10.1093/rap/rky021 30506023 PMC6251482

[28] Sanchez-Santos MT Garriga C Judge A et al. Development and validation of a clinical prediction model for patient-reported pain and function after primary total knee replacement surgery Sci Rep 2018 8 1 3381 10.1038/s41598-018-21714-1 29467465 PMC5821875

[29] Mu Y Edwards JR Horan TC Berrios-Torres SI Fridkin SK Improving risk-adjusted measures of surgical site infection for the National Healthcare Safely Network Infect Control Hosp Epidemiol 2011 32 10 970 986 10.1086/662016 21931247

[30] Berbari EF Osmon DR Lahr B et al. The Mayo prosthetic joint infection risk score: implication for surgical site infection reporting and risk stratification Infect Control Hosp Epidemiol 2012 33 8 774 781 10.1086/666641 22759544

[31] Bozic KJ Ong K Lau E et al. Estimating risk in Medicare patients with THA: an electronic risk calculator for periprosthetic joint infection and mortality Clin Orthop Relat Res 2013 471 2 574 583 10.1007/s11999-012-2605-z 23179112 PMC3549162

[32] Kunutsor SK Whitehouse MR Blom AW Beswick AD Systematic review of risk prediction scores for surgical site infection or periprosthetic joint infection following joint arthroplasty Epidemiol Infect 2017 145 9 1738 1749 10.1017/S0950268817000486 28264756 PMC9203287

[33] Mesko NW Bachmann KR Kovacevic D LoGrasso ME O’Rourke C Froimson MI Thirty-day readmission following total hip and knee arthroplasty - a preliminary single institution predictive model J Arthroplasty 2014 29 8 1532 1538 10.1016/j.arth.2014.02.030 24703364

[34] Paxton EW Inacio MCS Khatod M Yue E Funahashi T Barber T Risk calculators predict failures of knee and hip arthroplasties: findings from a large health maintenance organization Clin Orthop Relat Res 2015 473 12 3965 3973 10.1007/s11999-015-4506-4 26324831 PMC4626526

[35] Lungu E Desmeules F Dionne CE Belzile EL Vendittoli P-A Prediction of poor outcomes six months following total knee arthroplasty in patients awaiting surgery BMC Musculoskelet Disord 2014 15 1 299 10.1186/1471-2474-15-299 25201448 PMC4247215

[36] Huber M Kurz C Leidl R Predicting patient-reported outcomes following hip and knee replacement surgery using supervised machine learning BMC Med Inform Decis Mak 2019 19 1 3 10.1186/s12911-018-0731-6 30621670 PMC6325823

[37] Pua Y-H Poon C-L Seah F-T et al. Predicting individual knee range of motion, knee pain, and walking limitation outcomes following total knee arthroplasty Acta Orthop 2019 90 2 179 186 10.1080/17453674.2018.1560647 30973090 PMC6461070

[38] Van Onsem S Van Der Straeten C Arnout N Deprez P Van Damme G Victor J A new prediction model for patient satisfaction after total knee arthroplasty J Arthroplasty 2016 31 12 2660 2667 10.1016/j.arth.2016.06.004 27506723

[39] Thuraisingam S Dowsey M Manski-Nankervis J-A et al. Developing prediction models for total knee replacement surgery in patients with osteoarthritis: statistical analysis plan Osteoarthr Cartil Open 2020 2 4 100126 10.1016/j.ocarto.2020.100126 36474876 PMC9718256

[40] Buirs LD Van Beers LWAH Scholtes VAB Pastoors T Sprague S Poolman RW Predictors of physical functioning after total hip arthroplasty: a systematic review BMJ Open 2016 6 9 e010725 10.1136/bmjopen-2015-010725 27601486 PMC5020746

[41] Moher D Shamseer L Clarke M et al. Preferred reporting items for systematic review and meta-analysis protocols (PRISMA-P) 2015 statement Syst Rev 2015 4 1 1 10.1186/2046-4053-4-1 25554246 PMC4320440

[42] Shamseer L Moher D Clarke M et al. Preferred reporting items for systematic review and meta-analysis protocols (PRISMA-P) 2015: elaboration and explanation BMJ 2015 349 g7647 10.1136/bmj.g7647 25555855

[43] Chang WJ Naylor J Natarajan P Liu V Adie S Evaluating methodological quality of prognostic prediction models on patient reported outcome measurements after total hip replacement and total knee replacement surgery: a systematic review protocol Syst Rev 2022 11 1 165 10.1186/s13643-022-02039-7 35948989 PMC9364604

[44] Debray TPA Damen JAAG Snell KIE et al. A guide to systematic review and meta-analysis of prediction model performance BMJ 2017 356 i6460 10.1136/bmj.i6460 28057641

[45] Harris K Dawson J Gibbons E et al. Systematic review of measurement properties of patient-reported outcome measures used in patients undergoing hip and knee arthroplasty Patient Relat Outcome Meas 2016 7 101 108 10.2147/PROM.S97774 27524925 PMC4966645

[46] Ware J Kosinski M Keller SD A 12-item Short-Form Health survey: construction of scales and preliminary tests of reliability and validity Med Care 1996 34 3 220 233 10.1097/00005650-199603000-00003 8628042

[47] Ware JE Sherbourne CD The MOS 36-item short-form health survey (SF-36). I. Conceptual framework and item selection Med Care 1992 30 6 473 483 1593914

[48] Rabin R de Charro F EQ-5D: a measure of health status from the EuroQol Group Ann Med 2001 33 5 337 343 10.3109/07853890109002087 11491192

[49] Scuderi GR Bourne RB Noble PC Benjamin JB Lonner JH Scott WN The New Knee Society Knee Scoring System Clin Orthop Relat Res 2012 470 1 3 19 10.1007/s11999-011-2135-0 22045067 PMC3237971

[50] Bellamy N Buchanan WW Goldsmith CH Campbell J Stitt LW Validation study of WOMAC: a health status instrument for measuring clinically important patient relevant outcomes to antirheumatic drug therapy in patients with osteoarthritis of the hip or knee J Rheumatol 1988 15 12 1833 1840 3068365

[51] Dawson J Fitzpatrick R Murray D Carr A Questionnaire on the perceptions of patients about total knee replacement J Bone Joint Surg Br 1998 80-B 1 63 69 10.1302/0301-620x.80b1.7859 9460955

[52] Murray DW Fitzpatrick R Rogers K et al. The use of the Oxford hip and knee scores J Bone Joint Surg Br 2007 89-B 8 1010 1014 17785736 10.1302/0301-620X.89B8.19424

[53] Dawson J Fitzpatrick R Carr A Murray D Questionnaire on the perceptions of patients about total hip replacement J Bone Joint Surg Br 1996 78-B 2 185 190 8666621

[54] Rolfson O Bohm E Franklin P et al. Patient-reported outcome measures in arthroplasty registries: report of the patient-reported outcome measures working group of the International Society of Arthroplasty Registries Part II. Recommendations for selection, administration, and analysis Acta Orthop 2016 87 Suppl 1 Suppl 1 9 23 10.1080/17453674.2016.1181816 27228230 PMC4937770

[55] Moher D Liberati A Tetzlaff J Altman DG PRISMA Group Preferred reporting items for systematic reviews and meta-analyses: the PRISMA statement PLoS Med 2009 6 7 e1000097 10.1371/journal.pmed.1000097 19621072 PMC2707599

[56] Collins GS Reitsma JB Altman DG Moons KGM Transparent reporting of a multivariable prediction model for individual prognosis or diagnosis (TRIPOD): the TRIPOD statement Br J Surg 2015 102 3 148 158 10.1002/bjs.9736 25627261

[57] Steyerberg EW Vickers AJ Cook NR et al. Assessing the performance of prediction models: a framework for traditional and novel measures Epidemiology 2010 21 1 128 138 10.1097/EDE.0b013e3181c30fb2 20010215 PMC3575184

[58] Moons KGM Wolff RF Riley RD et al. PROBAST: a tool to assess risk of bias and applicability of prediction model studies: explanation and elaboration Ann Intern Med 2019 170 1 W1 W33 10.7326/M18-1377 30596876

[59] Berliner JL Brodke DJ Chan V SooHoo NF Bozic KJ Preoperative patient-reported outcome measures predict clinically meaningful improvement in function after THA Clin Orthop Relat Res 2016 474 2 321 329 10.1007/s11999-015-4350-6 26201420 PMC4709271

[60] Berliner JL Brodke DJ Chan V SooHoo NF Bozic KJ Can preoperative patient-reported outcome measures be used to predict meaningful improvement in function after TKA? Clin Orthop Relat Res 2017 475 1 149 157 10.1007/s11999-016-4770-y 26956248 PMC5174023

[61] Clement ND Weir DJ Holland J Deehan DJ Is there a threshold preoperative WOMAC score that predicts patient satisfaction after total knee arthroplasty? J Knee Surg 2021 34 8 846 852 10.1055/s-0039-3401826 31830763

[62] Farooq H Deckard ER Ziemba-Davis M Madsen A Meneghini RM Predictors of patient satisfaction following primary total knee arthroplasty: results from a traditional statistical model and a machine learning algorithm J Arthroplasty 2020 35 11 3123 3130 10.1016/j.arth.2020.05.077 32595003

[63] Harris AHS Kuo AC Bowe TR Manfredi L Lalani NF Giori NJ Can machine learning methods produce accurate and easy-to-use preoperative prediction models of one-year improvements in pain and functioning after knee arthroplasty? J Arthroplasty 2021 36 1 112 117 10.1016/j.arth.2020.07.026 32798181

[64] Judge A Javaid MK Arden NK et al. Clinical tool to identify patients who are most likely to achieve long‐term improvement in physical function after total hip arthroplasty Arthritis Care Res (Hoboken) 2012 64 6 881 889 10.1002/acr.21594 22232080

[65] Katakam A Karhade AV Collins A et al. Development of machine learning algorithms to predict achievement of minimal clinically important difference for the KOOS-PS following total knee arthroplasty J Orthop Res 2022 40 4 808 815 10.1002/jor.25125 34275163

[66] Kunze KN Polce EM Sadauskas AJ Levine BR Development of machine learning algorithms to predict patient dissatisfaction after primary total knee arthroplasty J Arthroplasty 2020 35 11 3117 3122 10.1016/j.arth.2020.05.061 32564970

[67] Lungu E Vendittoli PA Desmeules F Identification of patients with suboptimal results after hip arthroplasty: development of a preliminary prediction algorithm BMC Musculoskelet Disord 2015 16 279 10.1186/s12891-015-0720-1 26438322 PMC4595123

[68] Nemes S Rolfson O Garellick G Development and validation of a shared decision-making instrument for health-related quality of life one year after total hip replacement based on quality registries data J Eval Clin Pract 2018 24 1 13 21 10.1111/jep.12603 27461743

[69] Pronk Y Peters MCWM Brinkman J-M Is patient satisfaction after total knee arthroplasty predictable using patient characteristics and preoperative patient-reported outcomes? J Arthroplasty 2021 36 7 2458 2465 10.1016/j.arth.2021.02.064 33741243

[70] Pua Y-H Seah FJ-T Clark RA Poon CL-L Tan JW-M Chong H-C Development of a prediction model to estimate the risk of walking limitations in patients with total knee arthroplasty J Rheumatol 2016 43 2 419 426 10.3899/jrheum.150724 26628603

[71] Pua Y-H Poon CL-L Seah FJ-T et al. Predicting individual knee range of motion, knee pain, and walking limitation outcomes following total knee arthroplasty Acta Orthop 2019 90 2 179 186 10.1080/17453674.2018.1560647 30973090 PMC6461070

[72] Rogers BA Alolabi B Carrothers AD Kreder HJ Jenkinson RJ Can the pre-operative Western Ontario and McMaster score predict patient satisfaction following total hip arthroplasty? Bone Joint J 2015 97-B 2 150 153 10.1302/0301-620X.97B2.34718 25628274

[73] Sniderman J Stark RB Schwartz CE Imam H Finkelstein JA Nousiainen MT Patient factors that matter in predicting hip arthroplasty outcomes: a machine-learning approach J Arthroplasty 2021 36 6 2024 2032 10.1016/j.arth.2020.12.038 33558044

[74] Tanaka S Amano T Uchida S et al. A clinical prediction rule for predicting a delay in quality of life recovery at 1 month after total knee arthroplasty: a decision tree model J Orthop Sci 2021 26 3 415 420 10.1016/j.jos.2020.04.010 32507325

[75] Vissers LCM van Hove RP van der Zwaard BC Predicting self-reported functional improvement one year after primary total knee arthroplasty using pre- and postoperative patient-reported outcome measures Knee 2020 27 3 683 689 10.1016/j.knee.2020.04.006 32563423

[76] Wang W Morrison TA Geller JA Yoon RS Macaulay W Predicting short-term outcome of primary total hip arthroplasty:a prospective multivariate regression analysis of 12 independent factors J Arthroplasty 2010 25 6 858 864 10.1016/j.arth.2009.06.011 19679437

[77] Weber M Zeman F Craiovan B et al. Predicting outcome after total hip arthroplasty: the role of preoperative patient-reported measures Biomed Res Int 2019 2019 1 4909561 10.1155/2019/4909561 30834267 PMC6374818

[78] Aggarwal A Naylor JM Adie S Liu VK Harris IA Preoperative factors and patient-reported outcomes after total hip arthroplasty: multivariable prediction modeling J Arthroplasty 2022 37 4 714 720 10.1016/j.arth.2021.12.036 34990754

[79] Zhou Y Dowsey M Spelman T Choong P Schilling C SMART choice (knee) tool: a patient-focused predictive model to predict improvement in health-related quality of life after total knee arthroplasty ANZ J Surg 2023 93 1–2 316 327 10.1111/ans.18250 36637215

[80] Vervullens S Meert L Smeets RJEM et al. Preoperative glycaemic control, number of pain locations, structural knee damage, self-reported central sensitisation, satisfaction and personal control are predictive of 1-year postoperative pain, and change in pain from pre- to 1-year posttotal knee arthroplasty Knee Surg Sports Traumatol Arthrosc 2025 33 1 201 219 10.1002/ksa.12265 38751081 PMC11716348

[81] Zhang S Chen JY Pang HN Lo NN Yeo SJ Liow MHL Development and internal validation of machine learning algorithms to predict patient satisfaction after total hip arthroplasty Arthroplasty 2021 3 1 33 10.1186/s42836-021-00087-3 35236492 PMC8796459

[82] Strahl A Delsmann MM Simon A Ries C Rolvien T Beil FT A clinical risk score enables early prediction of dissatisfaction 1 year after total knee arthroplasty Knee Surg Sports Traumatol Arthrosc 2025 33 1 252 264 10.1002/ksa.12277 38796721 PMC11716356

[83] Dowsey MM Spelman T Choong PFM A nomogram for predicting non-response to surgery one year after elective total hip replacement J Clin Med 2022 11 6 1649 10.3390/jcm11061649 35329975 PMC8955143

[84] Langenberger B Schrednitzki D Halder AM Busse R Pross CM Predicting whether patients will achieve minimal clinically important differences following hip or knee arthroplasty: a performance comparison of machine learning, logistic regression, and pre-surgery PROM scores using data from nine German hospitals Bone Joint Res 2023 12 9 512 521 10.1302/2046-3758.129.BJR-2023-0070.R2 37652447 PMC10471446

[85] Bloomfield RA Broberg JS Williams HA Lanting BA McIsaac KA Teeter MG Machine learning and wearable sensors at preoperative assessments: functional recovery prediction to set realistic expectations for knee replacements Med Eng Phys 2021 89 14 21 10.1016/j.medengphy.2020.12.007 33608121

[86] Fontana MA Lyman S Sarker GK Padgett DE MacLean CH Can machine learning algorithms predict which patients will achieve minimally clinically important differences from total joint arthroplasty? Clin Orthop Relat Res 2019 477 6 1267 1279 10.1097/CORR.0000000000000687 31094833 PMC6554103

[87] Garriga C Sanchez-Santos MT Judge A et al. Development of a model predicting non-satisfaction 1 year after primary total knee replacement in the UK and transportation to Switzerland Sci Rep 2018 8 1 3380 10.1038/s41598-018-21713-2 29467402 PMC5821704

[88] Liu J Yang Y Wan S et al. A new prediction model for patient satisfaction after total knee arthroplasty and the roles of different scoring systems: a retrospective cohort study J Orthop Surg Res 2021 16 1 329 10.1186/s13018-021-02469-4 34016153 PMC8136158

[89] Stöckli C Theiler R Sidelnikov E et al. Validity of a simple Internet-based outcome-prediction tool in patients with total hip replacement: a pilot study J Telemed Telecare 2014 20 3 117 122 10.1177/1357633X13519040 24585892 PMC4509886

[90] Tolk JJ Waarsing JEH Janssen RPA van Steenbergen LN Bierma-Zeinstra SMA Reijman M Development of preoperative prediction models for pain and functional outcome after total knee arthroplasty using the Dutch arthroplasty register data J Arthroplasty 2020 35 3 690 698 10.1016/j.arth.2019.10.010 31711805

[91] Twiggs JG Wakelin EA Fritsch BA et al. Clinical and statistical validation of a probabilistic prediction tool of total knee arthroplasty outcome J Arthroplasty 2019 34 11 2624 2631 10.1016/j.arth.2019.06.007 31262622

[92] Calkins TE Culvern C Nahhas CR et al. External validity of a new prediction model for patient satisfaction after total knee arthroplasty J Arthroplasty 2019 34 8 1677 1681 10.1016/j.arth.2019.04.021 31056443

[93] Pulik Ł Jaśkiewicz K Sarzyńska S Małdyk P Łęgosz P Modified frailty index as a predictor of the long-term functional result in patients undergoing primary total hip arthroplasty Reumatologia 2020 58 4 213 220 10.5114/reum.2020.98433 32921828 PMC7477476

[94] Riddle DL Golladay GJ Jiranek WA Perera RA External validation of a prognostic model for predicting nonresponse following knee arthroplasty J Arthroplasty 2017 32 4 1153 1158 10.1016/j.arth.2016.11.007 27919582 PMC5362316

[95] Chen L Overview of clinical prediction models Ann Transl Med 2020 8 4 71 10.21037/atm.2019.11.121 32175364 PMC7049012

[96] Helmrich IRAR Mikolić A Kent DM et al. Does poor methodological quality of prediction modeling studies translate to poor model performance? An illustration in traumatic brain injury Diagn Progn Res 2022 6 1 8 10.1186/s41512-022-00122-0 35509061 PMC9068255

[97] Gu J Chen R Wang SM et al. Does poor methodological quality of prediction modeling studies translate to poor model performance? An illustration in traumatic brain injury Cancer Prev Res (Phila) 2022 15 5 309 318 10.1158/1940-6207.CAPR-21-0426 35509061 PMC9068255

[98] Janssen KJM Donders ART Harrell FE et al. Missing covariate data in medical research: to impute is better than to ignore J Clin Epidemiol 2010 63 7 721 727 10.1016/j.jclinepi.2009.12.008 20338724

[99] Dhiman P Ma J Qi C et al. Sample size requirements are not being considered in studies developing prediction models for binary outcomes: a systematic review BMC Med Res Methodol 2023 23 1 188 10.1186/s12874-023-02008-1 37598153 PMC10439652

[100] Mallett S Royston P Dutton S Waters R Altman DG Reporting methods in studies developing prognostic models in cancer: a review BMC Med 2010 8 20 10.1186/1741-7015-8-20 20353578 PMC2856521

[101] Bouwmeester W Zuithoff NPA Mallett S et al. Reporting and methods in clinical prediction research: a systematic review PLoS Med 2012 9 5 1 12 10.1371/journal.pmed.1001221 22629234 PMC3358324

[102] EQUATOR Network Introducing the Transparent Reporting of a Multivariable Prediction Model for Individual Prognosis or Diagnosis Initiative: The TRIPOD Statement. Oxford: EQUATOR Network https://www.equator-network.org/reporting-guidelines/tripod-statement/ date last accessed 9 December 2025

[103] Simera I Moher D Hirst A Hoey J Schulz KF Altman DG Transparent and accurate reporting increases reliability, utility, and impact of your research: reporting guidelines and the EQUATOR Network BMC Med 2010 8 1 24 10.1186/1741-7015-8-24 20420659 PMC2874506

[104] Gagnier JJ Moher D Boon H Beyene J Bombardier C Investigating clinical heterogeneity in systematic reviews: a methodologic review of guidance in the literature BMC Med Res Methodol 2012 12 1 5 10.1186/1471-2288-12-111 22846171 PMC3564789

[105] Collins GS Moons KGM Dhiman P TRIPOD+AI statement: updated guidance for reporting clinical prediction models that use regression or machine learning methods BMJ 2024 e078378 10.1136/bmj-2023-078378 38626948 PMC11019967

[106] Collins GS Dhiman P Andaur Navarro CL et al. Protocol for development of a reporting guideline (TRIPOD-AI) and risk of bias tool (PROBAST-AI) for diagnostic and prognostic prediction model studies based on artificial intelligence BMJ Open 2021 11 7 e048008 10.1136/bmjopen-2020-048008 34244270 PMC8273461

[107] Fihn SD Berlin JA Haneuse SJPA Rivara FP Prediction models and clinical outcomes—a call for papers JAMA Netw Open 2024 7 4 e249640 10.1001/jamanetworkopen.2024.9640 38607631

